# Expression of *Pennisetum glaucum* Eukaryotic Translational Initiation Factor 4A (*PgeIF4A*) Confers Improved Drought, Salinity, and Oxidative Stress Tolerance in Groundnut

**DOI:** 10.3389/fpls.2017.00453

**Published:** 2017-04-07

**Authors:** Tata Santosh Rama Bhadra Rao, Juturu Vijaya Naresh, Palakolanu Sudhakar Reddy, Malireddy K. Reddy, Garladinne Mallikarjuna

**Affiliations:** ^1^Plant Molecular Biology Laboratory, Agri Biotech FoundationHyderabad, India; ^2^Cell, Molecular Biology and Genetic Engineering Group, International Crops Research Institute for the Semi-Arid TropicsHyderabad, India; ^3^Crop improvement group, International Center for Genetic Engineering and BiotechnologyNew Delhi, India

**Keywords:** de-embryonated cotyledons, *nos-bar*, *rd29A*, *PgeIF4A*, JL-24, segregation, transcript analysis

## Abstract

Eukaryotic translational initiation factor 4A belong to family of helicases, involved in multifunctional activities during stress and non-stress conditions. The *eIF4A* gene was isolated and cloned from semi-arid cereal crop of *Pennisetum glaucum*. In present study, the *PgeIF4A* gene was expressed under the regulation of stress inducible *Arabidopsis rd29A* promoter in groundnut (*cv* JL-24) with *bar* as a selectable marker. The de-embryonated cotyledons were infected with *Agrobacterium tumefaciens* (LBA4404) carrying *rd29A:PgeIF4A* construct and generated high frequency of multiple shoots in phosphinothricin medium. Twenty- four T_0_ plants showed integration of both *nos-bar* and *rd29A-PgeIF4A* gene cassettes in genome with expected amplification products of 429 and 654 bps, respectively. Transgene copy number integration was observed in five T_0_ transgenic plants through Southern blot analysis. Predicted Mendelian ratio of segregation (3:1) was noted in transgenic plants at T_1_ generation. The T_2_ homozygous lines (L1-5, L8-2, and L16-2) expressing *PgeIF4A* gene were exhibited superior growth performance with respect to phenotypic parameters like shoot length, tap root length, and lateral root formation under simulated drought and salinity stresses compared to the wild type. In addition, the chlorophyll retention was found to be higher in these plants compared to the control plants. The quantitative real time—PCR results confirmed higher expression of *PgeIF4A* gene in L1-5, L8-3, and L16-2 plants imposed with drought/salt stress. Further, the salt stress tolerance was associated with increase in oxidative stress markers, such as superoxide dismutase accumulation, reactive oxygen species scavenging, and membrane stability in transgenic plants. Taken together our results confirmed that the *PgeIF4A* gene expressing transgenic groundnut plants exhibited better adaptation to stress conditions.

## Introduction

Groundnut or peanut (*Arachis hypogea* L.) is one of the important food legumes and oil seed crops grown in the semi-arid tropics of the world. It is being cultivated on over 25.2 million ha worldwide with a total production of 41.2 million tons with an average yield of 1.67 tons/ha. India is the second largest producer of groundnut accounting 8 million tons from 6 million ha (FAOSTAT, 2014) which needs to be increased up to 14.8 million tons by 2020 to meet the growing demand. Most of the cultivated groundnut varieties are highly sensitive to abiotic and biotic stresses such as drought, salinity, low temperature, insects, fungal, viral, and bacterial diseases (Kumar and Kirti, 2015).

Conventional breeding approaches for abiotic stress tolerance in groundnut has met with limited success due to non-availability of desired QTLs and levels of polymorphisms in cultivated varieties. As groundnut is self-pollinating crop, transfer of genes into sensitive popular varieties through marker-assisted selection may leads to linkage drag (Bhatnagar-Mathur et al., 2014). An alternative, genetic engineering approach offers novel solution by transferring alien genes for improving groundnut varieties for abiotic stress tolerance.

Different classes of genes related to abiotic stress have been used to express in groundnut for improvement of stress tolerance. Overexpression of *AtNHX* (*Na*^+^*/H*^+^ *antiporter*) gene in groundnut leads to compartmentalization of Na^+^ ions in vacuoles, thereby improved tolerance against salinity and limited water conditions (Asif et al., 2011). A bacterial *mtlD* gene encoding *mannitol 1-phosphate dehydrogenase* when expressed in groundnut cultivar GG20 under a constitutive promoter *CaMV35s* conferred drought tolerance through accumulation of mannitol (Bhauso et al., 2014). The genes from *Salicornia brachiata, ascorbate peroxidase* (*SbAPX*), and *abscisic acid stress ripening* (*SbASR*) genes when expressed in groundnut displayed salt and drought stress tolerance coupled with more chlorophyll retention and enhanced levels of relative water content under normal/stress conditions (Singh et al., 2014; Tiwari et al., 2015). The transcription factors from *Arabidopsis, AtNAC2* (Patil et al., 2014), *AtDREB1A* (Sarkar et al., 2014, 2016) and horsegram, *MuNAC4* (Pandurangaiah et al., 2014) were exhibited drought and salt stress tolerance in groundnut by reducing the membrane damage and improving scavenging of reactive oxygen species scavenging (ROS). Groundnut transgenic plants (*cv* TMV-2) simultaneously expressing three TFs (*AtDREB2A, AtHB7*, and *AtABF3*) displayed to increase drought, salinity, and oxidative stress tolerance compared to wild type (Pruthvi et al., 2014). The *AtDREB1A* transcription factor was expressed in groundnut under the regulation of either *rd29A* or *CaMV35s* promoter separately. The phenotype of *35s:AtDREB1A* exhibited delay in germination with severe growth retardation. However, positive growth regulation was seen in *rd29A:AtDREB1A* expressing plants and thereby improved 40% transpiration efficiency under limited water conditions (Bhatnagar-Mathur et al., 2014). Thus, expression of the stress related genes under the regulation of stress inducible promoters could be more rewarding rather than constitutive expression to minimize negative effects on plant growth performance (Sarkar et al., 2016).

Eukaryotic translational initiation factor 4A proteins belong to a family of DEAD box DNA/RNA helicases which catalyze the unwinding of stable RNA or DNA duplex during translation process. *eIF4A* is a prototype member of DEAD box helicase family protein that mediates the unwinding of double stranded RNA and binds to 40s ribosomal subunit during translational initiation process. Further these proteins stimulate stress induced pathways which mediate the stress tolerance (Tuteja et al., 2014). The *eIF4A* participates in ATP-dependent unwinding of the mRNA (Rogers et al., 2002; Linder, 2006). Plant based *eIF4A* was first reported in association with a cyclin dependent kinase during active cell proliferation (Hutchins et al., 2004). The *eIF4A* genes associated with abiotic stress tolerance have been identified from several plant species like tobacco (Owtrim et al., 1991), rice (Nishi et al., 1993), pea (Pham et al., 2000; Vashisht et al., 2005), and wheat (Metz and Browning, 1993).

The role of *eIF4A* genes in abiotic stress tolerance was studied through transgenic approach. A pea DNA helicase *(PDH45)* homologous to *eIF4A* when expressed in tobacco and rice imparted salt stress tolerance with minimal accumulation of sodium in transgenic plants (Sanan-Mishra et al., 2005; Nath et al., 2015). *PDH45* gene when expressed in groundnut variety (K-134) showed high tolerance against moisture stress (Manjulatha et al., 2014). Multiple stress responsive genes such as *OsAlfin* (alfalfa zinc finger), *PDH45* and *PgHSF4* (*Pennisetum glaucum* heat shock factor) simultaneously expressed in groundnut conferred tolerance to simulated drought stress and enhanced yield (Ramu et al., 2015). Constitutive expression of *PDH45* gene along with *EaDREB2* was conferred higher salinity tolerance in sugarcane (Augustine et al., 2015).

Pearl millet [*Pennisetum glaucum* (L.) R. Br.], a hardy C4 plant, exhibits better adaptation to harsh arid or semi-arid climates as compared to other cereals (Yadav, 2010), thereby making it an ideal depository of several stress regulatory genes. In this study, we have cloned the cDNA encoding for *eIF4A* gene of *P. glaucum* and transferred into JL-24 variety through *Agrobacterium*. The transgenic groundnut plants were evaluated for their stress tolerance under different conditions and for their molecular and phenotypic features. This is the first report on evaluation and expression of *eIF4A* gene of *P. glaucum* in groundnut for revealing its role in drought and salinity stress tolerance.

## Materials and methods

### Cloning of *PgeIF4A* gene in binary vector under stress inducible promoter *rd29A*

The cDNA encoding for *PgeIF4A* gene was isolated from stress induced seedlings of *P. glaucum* by using homology based gene cloning approach. Degenerate primers were designed based on conserved sequences found in the closely related monocot species. The full length gene was amplified using specific primers *PgeIF4A*F *-Nde* I and *PgeIF4A*R -*Not* I. The *rd29A* promoter (Accession no: AY973635) was isolated from *Arabidopsis* genomic DNA by using promoter specific primers overhang with restriction enzymes, *Kpn* I in sense primer and *Nde* I in anti-sense primer. The *PgeIF4A* gene fused in sense orientation at *Nde* I–*Not* I sites at downstream to the *rd29A* promoter (*Kpn* I–*Nde* I) and upstream to poly A terminator (*Not* I–*Sac* I) in plant expression vector, pGreen0229 (www.pgreen.ac.uk/) with *bar* (*bialophos aminotransferase* driven by *nopaline synthase* promoter) as a selectable marker. The recombinant *rd29A:PgeIF4A:Poly A* construct was then confirmed by restriction and sequence analysis.

### *Agrobacterium* transformation of *PgeIF4A* through electroporation

The pGreen0229 vector harboring *rd29A-PgeIF4A–poly A* cassette and *pSoup* were co-transformed into *Agrobacterium tumefaciens* (LBA4404) with following condition of 2.5 kV for 5 μS by multi- electroporator (Eppendorf International, Germany). Electroporated samples were immediately transferred into 1 ml YEM (yeast extract mannitol) liquid medium and incubated for 5 h at 28°C with 200 rpm. Then the cells were harvested and plated on YEM medium supplemented with 50 mg/L kanamycin, 20 mg/L rifampicin, and 50 mg/L streptomycin. The recombinant colonies were observed after 48 h of incubation at 28°C and confirmed the positive colonies by PCR.

### Seed sterilization and explants preparation

Seeds of groundnut (*cv* JL-24) were collected from International Crops Research Institute for the Semi Arid Tropics (ICRISAT), Patancheru, India. Seeds were surface sterilized with 0.1% mercuric chloride for 8 min, followed through rinsing with sterile water and soaked for 4 h in distilled water. The seed coat was peeled off and the cotyledons were sliced vertically to prepare de-embryonated half cotyledon (DEC) aseptically which was used for *Agrobacterium* transformation.

### *Agrobacterium* mediated groundnut transformation with *PgeIF4A* using DEC explants

Healthy half DECs were infected with *A. tumefaciens* (LBA4404) suspension culture (*OD*_600_ = 0.5–0.6) by gentle shaking for 10 min and dried them on sterile Whatman filter paper under aseptic conditions. Then, the explants were co-cultivated for 48 h on MS basal medium (Murashige and Skoog, 1962) + B_5_ vitamins (Gamborg et al., 1968) supplemented with 3% sucrose, solidified with 0.8% agar and 100 μM acetosyringone. Further, the explants were washed with cefotaxime (250 mg/L) for the suppression of *Agrobacterium*. The explants transferred to shoot induction medium supplemented with MS salts + B_5_ + 24.5 mg/L of benzylaminopurine (BAP) + 21.5 mg/L of 2, 4—Dichlorophenoxy acetic acid (2,4-D) + 5 mg/L phosphinothricin (PPT) and 3% sucrose at pH 5.8 solidified with 0.8% agar. Cultures were maintained at 26 ± 1°C at 16 h photoperiod with white fluorescent light intensity of 60 mE m^2^s^−1^. Resistant induced shoots from un-differentiated calli at the stage of 3–5 cm length were transformed to shoot elongation medium supplemented with BAP (2 mg/L) and allowed to grow further for another 2 weeks to attain shoot elongation up to 7–9 cm. Elongated shoots were sub cultured into rooting medium containing NAA (0.5 mg/L) + PPT (5 mg/L) incubated for about 15 days. After root formation, the plantlets were transferred to transgenic glasshouse for further hardening and grown them till attaining the maturity. Seeds were harvested from the putative transformed and control plants for further analysis.

### Molecular confirmation of transgenic plants

Genomic DNA from un-transformed control and transformed plants (T_0_) was isolated using modified CTAB method (Doyle and Doyle, 1987). Transgene (*rd29A-PgeIF4A*) and marker gene (*nos-bar*) cassettes were PCR amplified using primer sets *rd29A F1: PgeIF4A R1* and *nos F1: bar R1* respectively (Supplementary Table 1). The PCR reaction was performed using the thermal profile: one cycle of initial denaturation at 94°C for 3 min; 35 cycles with repetition of 94°C for 1 min (denaturation), 55°C for 1 min (annealing), 72°C for 1 min (extension). The 50 μl PCR reaction mixture contained 200 ng of genomic DNA, 5 μl 10 X Taq buffer, 1 μl dNTP (10 mM), 1 μl forward primer (150 ng), 1 μl reverse primer (150 ng), 1 μl Taq (5 U) DNA polymerase enzyme and rest all make up to 50 μl with milli Q water. The amplified products were electrophoressed on 1% agarose gel and visualized on gel documentation system (G-Box Syngene, UK).

### Copy number detection by southern blotting

Genomic DNA (20 μg) from control and transformed plants (T_0_) was digested using 30 U of *Xho* I (Fermentas life sciences), single cutter of T-DNA and non-cutter of *PgeIF4A* gene. The digested samples were size fractionated on agarose gel (0.8%) for 16 h at 30 V. The depurinated and denatured DNA fragments were subsequently transferred to positively charged Hybond N^+^ nylon membrane (GE Health Care, USA). Biotin labeled *PgeIF4A* gene was used as probe. Pre-Hybridization, washing and detection of transgene signals were carried out by following manufacturer's instructions (Biotin DNA labeling and chromogenic detection kit-Thermo Scientifics, USA). The seeds obtained from Southern positive were used for further transgene segregation analysis.

### Transgene segregation analysis

The T_1_ seeds were collected from transgene integrated plants and used for segregation analysis. The goodness of fit of the ratio was tested using *Chi*-square test. Gene specific primers were used for the amplification of coding region of *PgeIF4A*. Leaves of 2-week-old plants of control and transgenic lines (T_1_) were dipped in PPT (5 mg/L) solution and assessed the herbicide resistance after 10 days.

### Phenotypic characterization of transformed seedlings (T_2_) through drought and salinity stress assays

Mannitol and NaCl were used for simulating drought and salt stress conditions in transgenic plants at seedling stage. T_2_ seeds (L1-5, L8-3, and L16-2) were placed on half strength MS medium supplemented with mannitol (0, 200, and 300 mM) and NaCl (0, 100, and 200 mM), incubated for 15 days at 25 ± 2°C under 16 h light /8 h dark photo-period. Three seedlings constituted one biological replicate. The phenotypic growth parameters like shoot length, tap root length and number of lateral roots were recorded to evaluate the effect of drought and salinity stresses on transgenic lines. All experiments were repeated thrice and the data statistically analyzed.

### Leaf disc senescence assay

Leaf discs (six discs per each biological sample with 1.0 cm diameter) from fully expanded leaves of 1-month-old plants (T_2_ plants of lines L1-5, L8-3, and L16-2) were excised using cork borer. The discs were floated on 5 ml solution of 0 (distilled water as control), 200, 300 mM mannitol and 100, 200 mM NaCl to simulate drought and salinity stress, respectively. These discs were incubated for 4 days under continuous illumination at 26°C for leaf senescence assay. Then, the leaf discs were macerated using mortar and pestle and diluted them in 80% acetone. The absorbance of the samples was recorded at A_664_ and A_667_ using spectrophotometer. The chlorophyll A, B, and total amount were calculated as described by Arnon (1949). Experiment was carried out as triplicates and the data was analyzed statistically.

### RNA isolation, cDNA synthesis, and quantitative real—time PCR

The total leaf RNA isolated using Tri-reagent (Sigma Aldrich) from the transgenic T_2_ lines as per the manufacturer's instructions (Sigma Aldrich). The quality and quantity of total RNA was assessed by Nano spectrophotometer (GE Health Care, USA) and RNA gel electrophoresis. The total RNA (5 μg) was reverse transcribed to cDNA using oligo dT primer as per manufacturer's instructions (iScript cDNA synthesis kit). The quantitative-real time PCR (qRT-PCR) was performed in Realplex (Eppendort, Germany) real time system. The gene specific primers and reference gene primers were designed using Primer 3.0 software to amplify the product of *PgeIF4A* and *glucose 6 phosphate-1-dehydrogenase* (*G6PD*, housekeeping internal groundnut gene; Reddy et al., 2013). The qRT-PCR reaction was performed in total 10 μl reaction containing 5 μl of 2X SensiFAST™ SYBR No-ROX (Bioline, UK) mix, 400 nM of each primer (Eurofins Scientifics) 1.0 μl diluted cDNA and nuclease free water to make the final volume. The thermal cycles were as follows: 95°C for 10 min followed by 40 cycles at 95°C for 15 s and 61°C for 1 min. After the qRT-PCR reaction was completed, a melting curve was generated to analyze the specificity of each gene by increasing the temperature from 60 to 95°C. The samples collected from three independent plants with three technical replicates. The threshold cycle (Ct) values were normalized using *G6PD* reference gene. The expression levels of *PgeIF4A* transcripts in different transgenic plants grown under control and stress treated were analyzed using qBase plus software (ver: 2.4; Biogazelle, Belgium; Hellemans et al., 2007).

### Biochemical characterization of transgenic plants exposed to salinity stress

To determine the salinity induced oxidative stress, 1 month old control and transgenic plants (L1-5, L8-3, and L16-2) were exposed to NaCl (250 mM) treatment for 10 days and maintained them under controlled greenhouse conditions.

#### Assessment of superoxide dismutase (SOD) activity

The SOD activity was assayed by monitoring the percentage of photochemical reduction of nitro bluetetrazolium (NBT) in leaf tissues according to a protocol described by Beyer and Fridovich (1987). The reaction mixture consisting of methionine (30 mg/mL), NBT (1.41 mg/mL), and triton X—100 (0.5%). To this reaction mixture, 50 μg of protein and 10 μl of riboflavin (0.44 mg/mL) were added and immediately kept under cool fluorescent light (500 μmol. m^−2^. s^−1^) to perform the reaction. The photo reduction of NBT (blue formazan production) was measured at 560 nm using spectrophotometer.

#### Assessment of lipid peroxidation through TBARS assay

The lipid peroxidation was determined by measuring malondialdehyde (MDA) content in leaf tissues through TBARS (thio barbutaric acid reactive substances) assay. Hundred milligrams of leaf tissue was homogenized in 2 mL of 0.1% TCA and centrifuged at 10,000 rpm for about 10 min. To 1 mL of supernatant 2 mL of 20% TCA and 2 mL of 0.5% TBA was added and incubated in water bath at 95 °C for 30 min, immediately cooled on ice and then centrifuged at 10,000 rpm for 10 min. The absorbance was measured at 532 and 600 nm. The value of absorbance at 600 nm was subtracted from absorbance at 532 nm. The MDA content was calculated using extinction coefficient of 155 mM^−1^ cm^−1^ (Arnon, 1949).

#### Cell membrane stability/electrolyte leakage

Electrolyte leakage (EL) was determined by following the standard protocol described by Kumari et al. (2015). Briefly, the leaf discs of control and transgenics were washed thoroughly with distilled water and incubated in 20 mL of deionized water for 2 h at 25°C. Electrolyte extracts drained into medium was recorded as E1 using electro conductivity meter. Later, the leaf discs were boiled for 30 min, subsequently allowed the samples to cool down and total electrolyte leakage was recorded (E2). Similarly, the EL was measured from control plants also. Total EL (E2) was determined after autoclaving the samples. The relative electrolyte leakage was calculated as (E1/E2) × 100.

#### 3, 3′ diaminobenzidine (DAB) assay

*In vitro* localization of H_2_O_2_ was determined by DAB assay as described by Kumari et al. (2015). Leaves of control and transgenic plants were vacuum infiltrated in 1 mg/L freshly prepared DAB solution (pH 3.8) for about 10 min at room temperature. The samples were then placed under light until dark spots appeared. The stained leaves were fixed with 3:1:1 ethanol: acetic acid: glycerol (v/v) solution.

### Statistical analysis

The experiments were designed as triplicates (three biological samples) and data analyzed statistically. To perform one-way and two-way ANOVA analysis, CoStat version 6.204 statistical program package (Cohort Software, Monterey, CA, USA) were used. One-way ANOVA was carried out to test the difference between the transgenic lines (L1-5, L8-3, and L16-2) and control. Means were compared using Tukey-Kramer test and LSD at *P* ≤ 0.05 (5% level). Subsequently, two-way ANOVA was used to assess treatment (T), plant lines (L), and plant lines by-treatment interactions (L × T) in two different stress treatments (Mannitol and NaCl).

## Results

### Cloning and *in-silico* analysis of *PgeIF4A* gene

**Figure 1:**
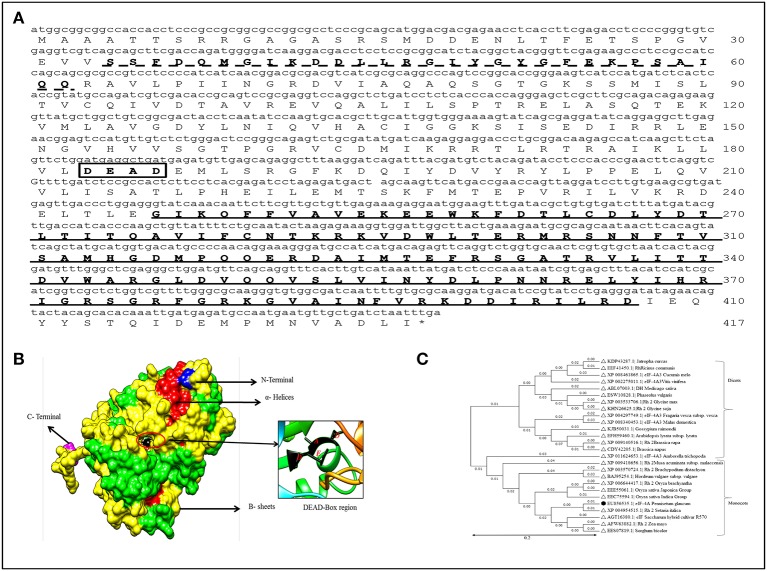
**Sequence analysis of *PgeIF4A* gene and protein. (A)** Schematic Representation of three motifs including RNA helicase/DEAD box Rec-Q-motif (34–62, indicated as dotted line), DEAD box domain (183–186, in box), and RNA-helicase C-terminal (246–407) in PgeIF4A protein. **(B)** Predicted three dimensional structure of PgeIF4A protein showing various domains in different colors, blue represented N-terminal region, magenta for C-terminal, red for α-Helices and green for β-sheets. DEAD box region highlighted in red color circle which harbor Asp-183 in β-5, Glu-184 in linked region and Ala-185, Asp-186 presented in α-9 regions. **(C)** Phylogenetic tree constructed based on deduced amino acid sequences of various eIF4A from closely related species. Protein names, accession numbers, and species names were indicated at each branch. The phylogenetic tree generated using Neighbor Joining (NJ) method and viewed using MEGA4 software.

The full length cDNA of *PgeIF4A* gene was isolated and cloned from *P. glaucum* using homology based cloning approach. The cloned gene sequence was deposited in the NCBI Genbank (Accession no: EU856535). The *PgeIF4A* gene consists of 1224 bp encoding for 407 amino acids with predicted molecular mass 45.2 kDa and isoelectric pH 6.10. The PgeIF4A protein consists of important motifs like RNA *helicase*/DEAD box Q-motif, DEAD box domain and *RNA-helicase* C-terminal (Figure 1A). These domains play major role in replication, recombination, splicing, ribosome biogenesis, RNA degradation, and repair mechanism. The domains of PgeIF4A protein were depicted in 3D modeling design. In this protein total 19 α-Helices and 13 β-sheets are present (Figure 1B). It was validated using UCSF-chimera, Rasmol, Phymol (Pettersen et al., 2004; Maheshwari and Brylinski, 2015). The phylogenetic analysis revealed that the protein belongs to the DEAD box helicase and shared highly conserved regions with *Setaria italica* (Figure 1C).

### Generation of *PgeIF4A* expressing groundnut transgenic plants

**Figure 2:**
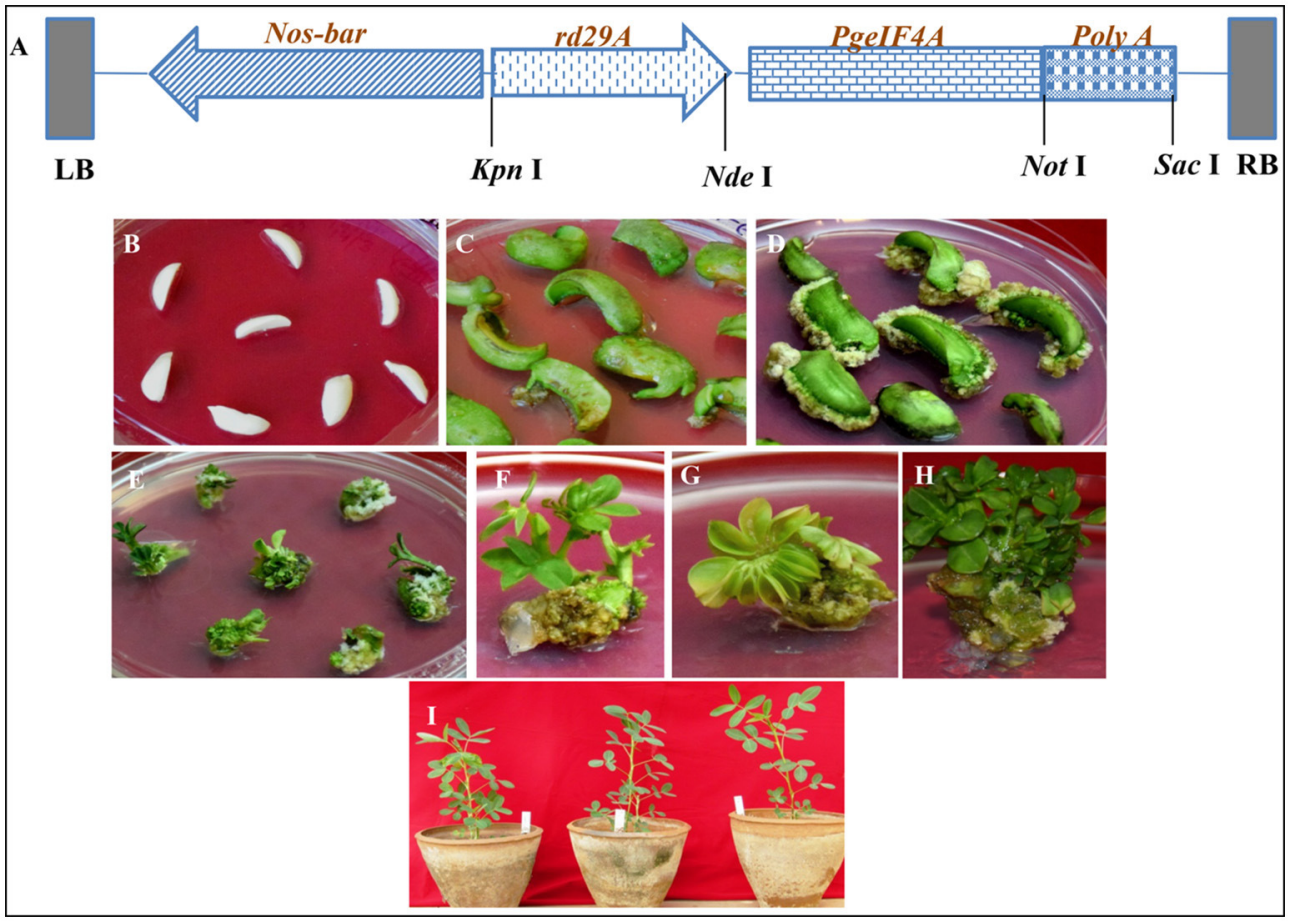
**Schematic representation of T-DNA region of recombinant plant transformation vector pGreen0229-*rd29A*:*PgeIF4A*:*poly A* and groundnut transformation. (A)** *PgeIF4A* gene was cloned under stress inducible promoter *rd29A* and *poly A* terminator, *bar* (*Bialaphos amino transferase* gene) as a selectable marker which driven by *nos* (*nopaline synthase*) promoter and *nos-*terminator. LB, left boarder; RB, right boarder. **(B)** De-embryonated half cotyledons (DEC) prepared from matured seeds. **(C,D)** DEC producing calli. **(E)** Phosphinothricin resistant calli producing shoots. **(F–H)** Shoot induction and multiple shoot formation. **(I)** Putative transgenic plants (T_0_) growing in glass house.

The *Agrobacterium tumefaciens* (LBA4404) carrying vector, pGreen0229: *rd29A: PgeIF4A: poly A* with *nos-bar* gene as selectable marker (Figure 2A) was used to infect 742 DECs (Figure 2B) prepared from matured groundnut seeds. Out of these 225 explants induced calli in the medium supplemented with phosphinothricin (PPT), BAP and 2, 4-D with a frequency of 30.3% (Figures 2C–E; Table 1). Subsequently these proliferated resistant calli differentiated into shoots in 2 weeks. About 38 green shoots from resistant calli (16.8%) differentiated into multiple shoots in 30–40 days (Figures 2F–H). The PPT resistant shoots were continuously grown and maintained on selective media. Roots generated on NAA supplemented medium. About 30 rooted plants were acclimatized under controlled greenhouse conditions (Figure 2I). The hardened plants grew normally and set flowers and pods. These plants were used for further molecular analysis.

**Table 1:** **Genetic transformation and regeneration frequency of peanut DEC explants of groundnut *cv* JL-24**.

**Explants infected with *Agrobacterium***	**Phosphinothricin resistant calli**	**Shoots regenerated from resistant calli**	**Rooted plants acclimatized in the soil/fertile plants**	**PCR positive plants**
742	225 (30.3%)	38 (16.8%)	30 (79%)	24 (11%)

### Molecular confirmation of *PgeIF4A* transgene integration

**Figure 3:**
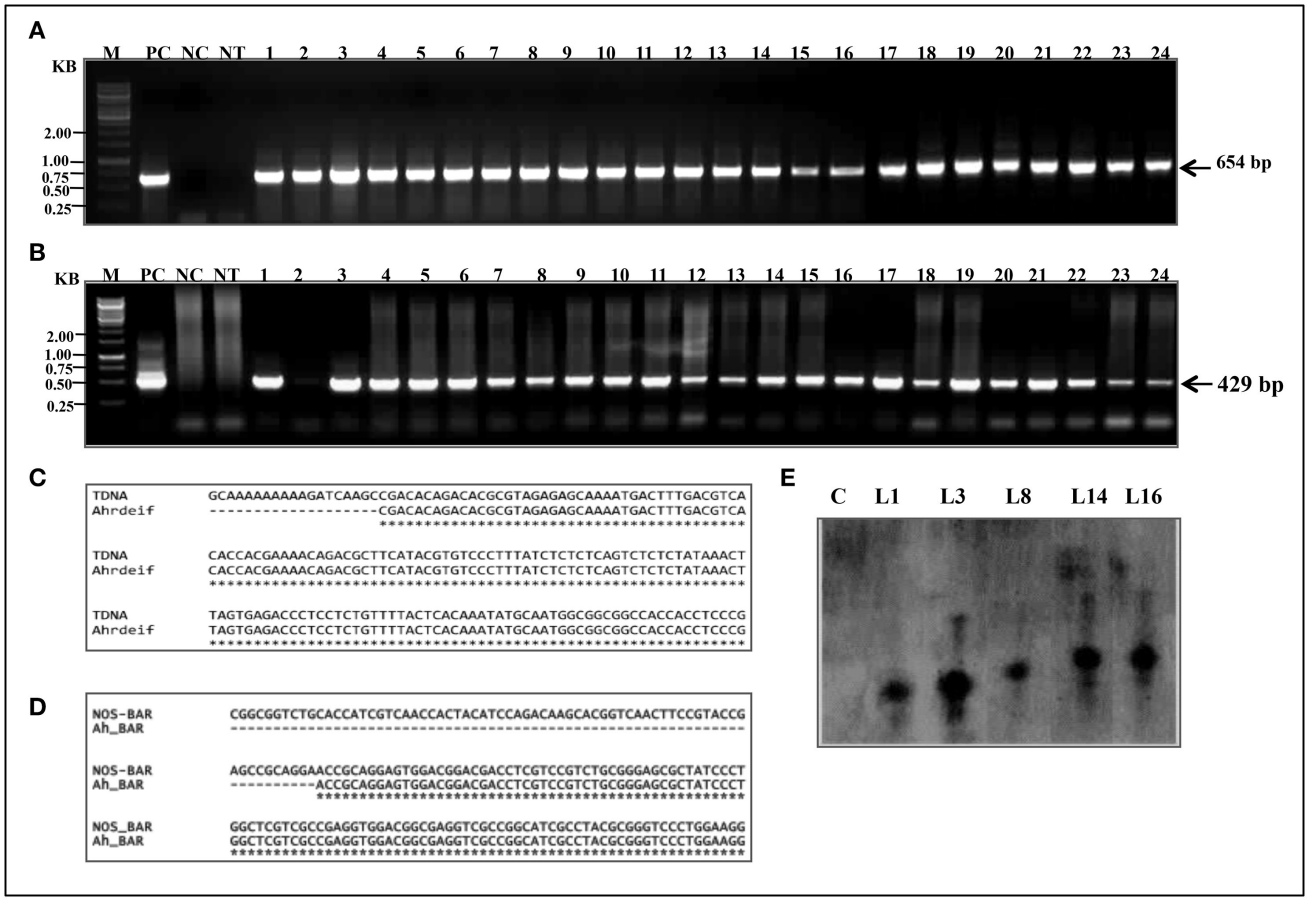
**Molecular confirmation of putative transgenic groundnut plants (T_0_). (A)** PCR amplification representing the junction of *rd29A-PgeIF4A* (654bp). **(B)** Junction of *nos-bar* marker gene. M, 1 kb marker; PC, positive control (plasmid DNA); NC, negative control; NT, non-transgenic plant; lanes 1–24, transgenic plant samples. **(C,D)** Alignment of nucleotide sequences of PCR amplified products *Ah_rdeIF*) and *Ah_BAR* from transgenic plants with recombinant vector pGreen0229-*PgeIF4A* sequence. **(E)** Depicted picture showing the analysis of Southern blot hybridization. The genomic DNA (20 μg) from control and transformed lines were digested with *Xho* I, size fractionated on 0.8% agarose gel, transferred to nylon membrane (Hybond N^+^), hybridized with biotin labeled *eIF4A* probe and detection of chromogenic signal was mediated through alkaline phosphatase reaction. Lane C, non-transgenic plant; Lane L1, L3, L8, L14, and L16 transgenic (T_0_) plants.

The PCR was performed for detecting the presence of *PgeIF4A* and *bar* gene cassettes at T_0_ generation in putative transgenic plants. Out of 30 phosphinothricin resistant (T_0_) plants, 24 showed amplification of expected 654 bp (Figure 3A) and 429 bp (Figure 3B) fragments of gene and marker gene cassettes, respectively. The products were amplified by using *rd29A* F1-*PgeIF4A* R1 (*rd29A* promoter-*eIF4A* gene junction) and *nos* F1-*bar* R1 (*nos*-*bar* junction) primer sets (Supplementary Table 1), respectively. The primers were designed one from promoter region another from gene region to avoid non-specific amplification of products. However, control plants failed such amplification of neither of them. The transformation frequency of JL-24 transgenics was noted as 11% based on PCR results. Both PCR amplified *nos-bar* and *rd29A-PgeIF* PCR products from transgenic plants were further sequenced using Sanger's dideoxy method (Eurofins Genomics India) and annotated the sequences using multiple sequence alignment software (http://www.ebi.ac.uk/Tools/msa/clustalw2/). Both gene and marker sequences were integrated in groundnut genome exactly matched with vector T-DNA (Figures 3C,D). It indicated that the transgene was integrated in JL-24 plants.

### Southern blotting analysis and segregation analysis

The gene integration and copy number of *PgeIF4A* into genome of transgenic lines were confirmed by Southern blotting using *PgeIF4A* gene as a probe. Among 16 plants tested, five plants showed gene integration. In five, L1 and L8 showed single copy integration, whereas L3, L14, and L16 exhibited thick and overlapping band, which could be due to integration of two copies in these three plants (Figure 3E). However, no hybridization signal was observed in non-transgenic plants. BLASTn analysis of *PgeIF4A* probe sequence used in Southern hybridization did not show any similarity with *Arachis hypogea* native gene. It confirmed that the heterologous transgene (*PgeIF4A*) was integrated into groundnut transgenic plants.

**Figure 4:**
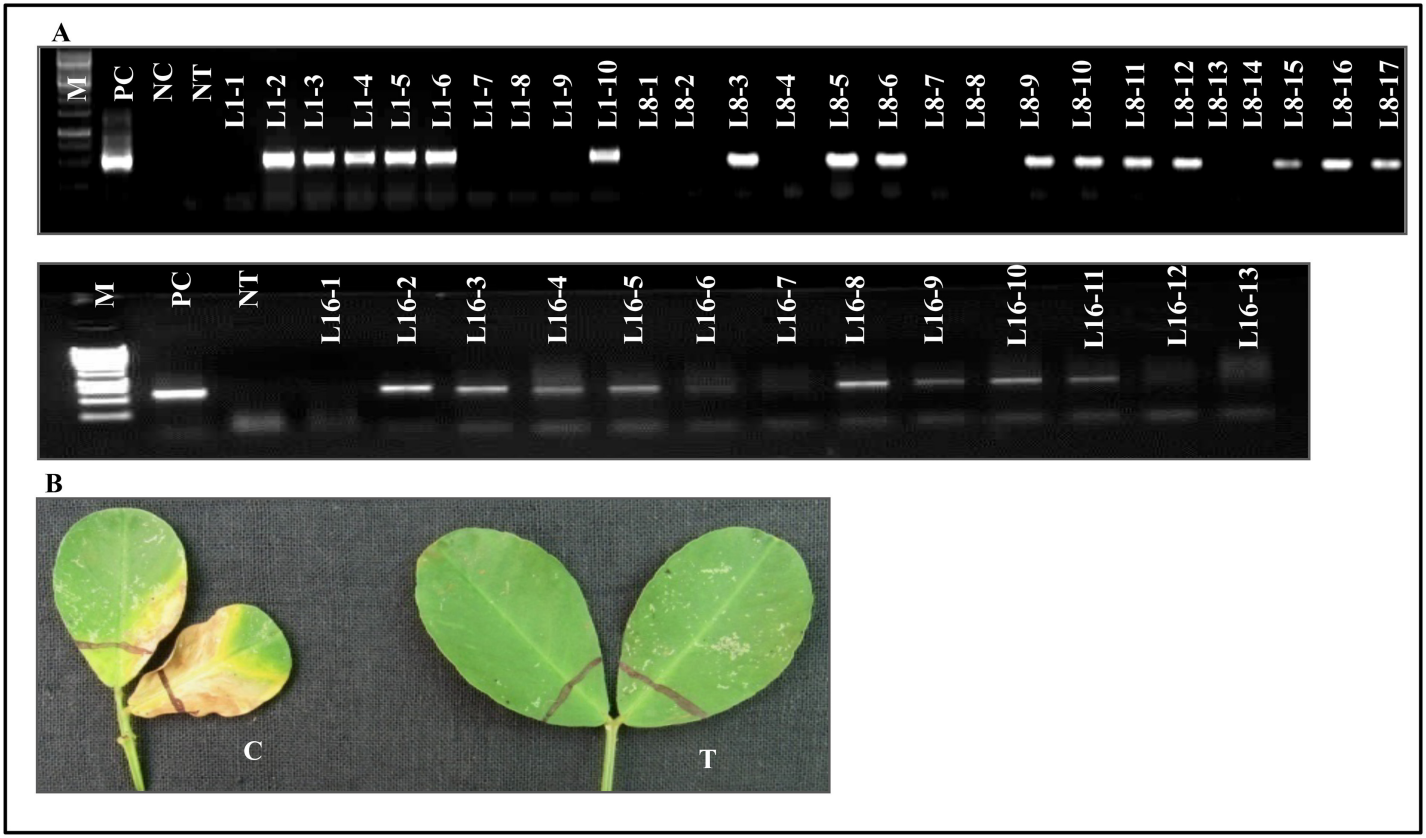
**Transgene segregation analysis. (A)** Depicted picture showing PCR amplification of *eIF4A* gene at T_1_ generation. L1-1 to L1-10, L8-1 to L8-17, and L16-1-L16-13 represents progeny of L1, L8, and L16 (T_0_) respectively. **(B)** Effect of PPT on leaves of groundnut transformants showing resistance to herbicide. C, wild type control leaf bleaching to herbicide; T, transgenic showing resistance to the herbicide.

T_1_ progeny seeds collected from single copy integrated southern positive (T_0_) plants were raised in glass house to determine the inheritance pattern of the transgene. The T_1_ progeny of L1, L8, and L16 was tested with PCR to detect the transgene. These lines exhibited the predicted Mendelian ratio as 3:1 (Figure 4A and Table 2). Simultaneously, all these T_1_ plants were again checked for their herbicide tolerance through PPT dip assay. The PCR positive plants showed the herbicide tolerance even after 10 days of exposure to PPT (Figure 4B). However, the control leaves were completely bleached out and wilted. It indicated that the transgenic plants expressed the *bar* gene and co segregated in the T_1_ generation which also followed the Mendelian law of segregation.

**Table 2:** **Segregation analysis of T_1_ transgenic plants expressing *PgeIF4A* gene**.

**Plant no**	**Total number of T_1_ plants**	**Number of PCR positive**	**χ^2^ value plants**	***P***
L1	10	6	1.2	0.27
L8	17	10	2.372	0.12
L16	13	9	0.23	0.631

*L1, L8, and L16 represents T_1_transgenic plants expressing PgeIF4A gene. The data of transgenic plants were analyzed using Chi square test. It showed the value <3.84 (p < 0.05) at 1° of freedom which was indicated good Mendelian segregation pattern in transgenic plants*.

### Characterization of *PgeIF4A* transgenic plants under simulated drought stress

**Figure 5:**
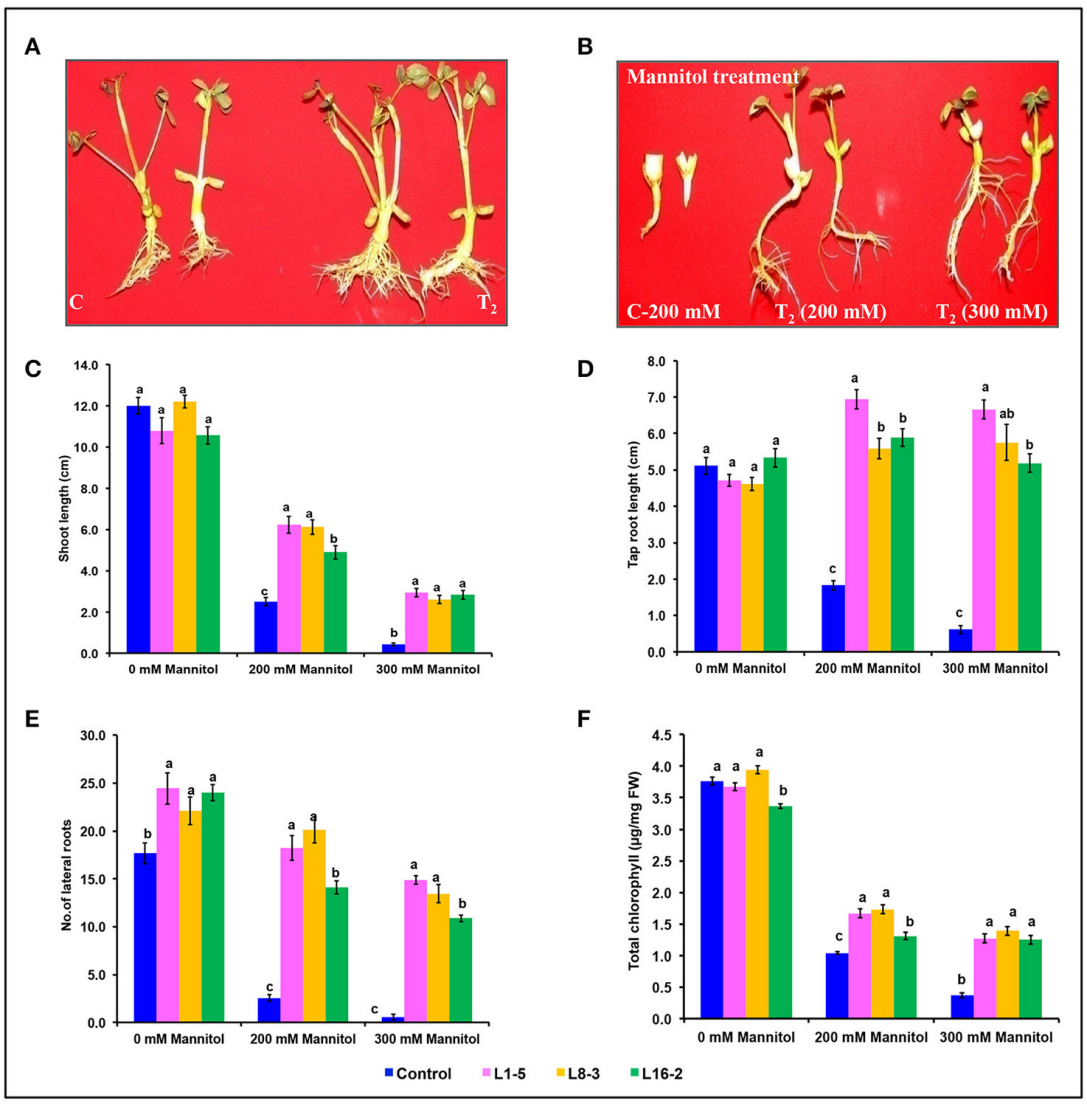
**Characterization of *eIF4A* groundnut transgenic plants under simulated drought stress conditions. (A)** Growth performance of *PgeIF4A* expressing transgenic plants (T_2_) and control (C) on plain MS medium. **(B)** Graphical representation of growth performance transgenic plants (T_2_) and control (C) on 200 and 300 mM mannitol. **(C)** Relative shoot length in cm. **(D)** Tap root length in cm. **(E)** Number of lateral roots. **(F)** Chlorophyll retention of transgenic and control plants grown under 0, 200, and 300 mM mannitol medium. All the experiments performed in triplicates and data represented as mean (*n* = three biological triplicates) using two way ANOVA with LSD and *P* < 0.005. Different alphabets indicated the significant differences between the treatments. Similar letters denotes non significant.

The transformed plants germinated normally without growth penalty like control plants (Figures 5A,B). There was a significant difference in the seedling growth of the wild type and transgenic lines under stress conditions. The shoot length and tap root length of transgenics exhibited higher growth than the wild type plants. Interestingly, the tap root system of transgenics treated with mannitol developed better than the control plants grown under normal conditions at both concentrations of mannitol (200 and 300 mM; Figures 5C,D; Tables 3, 4). The lateral root formation was completely inhibited in control, whereas these roots enormously proliferated under both 200 and 300 mM mannitol concentrations in case of transgenic lines (Figure 5E; Tables 3, 4).

**Table 3:** **Shoot length, tap root length, number of lateral roots, and total chlorophyll content of three transgenic plants and one control were subjected to different concentrations of mannitol stress treatments**.

**Traits**	**Components**	**Degree of freedom**	**Sum Square (SS)**	**Mean Square (MS)**	***F*-value**	***P-*value**	**Significance**
Shoot Length (cm)	Treatment (T)	2	2136.32	1068.16	778.47	0.000	^***^
Shoot Length (cm)	Transgenic lines (L)	3	83.18	27.73	20.21	0.000	^***^
Shoot Length (cm)	LxT interaction	6	100.51	16.75	12.21	0.000	^***^
Tap root length (cm)	Treatment (T)	2	6.7	3.35	4.23	0.017	^*^
Tap root length (cm)	Transgenic lines (L)	3	274.09	91.36	115.24	0.000	^***^
Tap root length (cm)	LxT interaction	6	171.01	28.5	35.95	0.000	^***^
No. of lateral roots	Treatment (T)	2	3682.18	1841.09	150.36	0.000	^***^
No. of lateral roots	Transgenic lines (L)	3	3487.4	1162.47	94.93	0.000	^***^
No. of lateral roots	LxT interaction	6	597.33	99.56	8.13	0.000	^***^
Total Chlorophyll	Treatment (T)	2	48.455	24.227	1818.97	0.000	^***^
Total Chlorophyll	Transgenic lines (L)	3	2.036	0.6787	50.955	0.000	^***^
Total Chlorophyll	LxT interaction	6	1.36	0.2267	17.022	0.000	^***^

*Two-way ANOVA was used to determine treatment (T), plant lines (L), and plant lines-by-treatment (LxT) effects*.

* and *** *Indicate significant and highly significant correlation at p < 0.05 and p < 0.01 respectively*.

**Table 4:** **Shoot length, tap root length, number of lateral roots, and total chlorophyll content of three transgenic plants and one control were subjected to mannitol stress treatments**.

**Traits**	**Treatment (mM-Mannitol)**	**Treatment mean**	**Significance**
**(A)**			
Shoot Length (cm)	0	11.38	A
Shoot Length (cm)	200	4.93	B
Shoot Length (cm)	300	2.19	C
Tap root length (cm)	0	5.06	A
Tap root length (cm)	200	4.94	AB
Tap root length (cm)	300	4.56	B
No. of lateral roots	0	22.06	A
No. of lateral roots	200	13.75	B
No. of lateral roots	300	9.94	C
Total Chlorophyll	0	3.694	A
Total Chlorophyll	200	1.437	B
Total Chlorophyll	300	1.07	C
**Traits**	**Plant lines**	**Mean of plants**	**Significance**
**(B)**			
Shoot Length (cm)	L1-5	6.97	A
Shoot Length (cm)	L8-3	6.64	AB
Shoot Length (cm)	L16-2	6.09	B
Shoot Length (cm)	Control	4.97	C
Tap root length (cm)	L1-5	6.1	A
Tap root length (cm)	L8-3	5.47	B
Tap root length (cm)	L16-2	5.32	B
Tap root length (cm)	Control	2.52	C
No. of lateral roots	L1-5	19.19	A
No. of lateral roots	L8-3	18.56	A
No. of lateral roots	L16-2	16.33	B
No. of lateral roots	Control	6.93	C
Total Chlorophyll	L1-5	2.353	A
Total Chlorophyll	L8-3	1.985	B
Total Chlorophyll	L16-2	2.206	A
Total Chlorophyll	Control	1.723	C

*Based on Two-way ANOVA results, treatments mean **(A)** and plant lines data **(B)** were compared by Turkey-Kramer test at 5% LSD. The treatments were significant, different alphabet letters were indicated. Similar letters denotes non-significant*.

In leaf senescence assay, on 2nd day of incubation, the leaf discs from control had bleaching of the chlorophyll with increasing concentration of mannitol. However, minimal bleaching was observed in leaf discs from transgenic plants which stayed green like the controls without stress. Chlorophyll A, B and total chlorophyll content of these leaf discs after 4 days of stress imposition retained 1.9–2.3 μg/mg FW, when compared to 1.7 μg/mg FW in control. However, there was no significant difference observed in chlorophyll content, when the concentration of mannitol increased from 200 to 300 mM (Figure 5F). The results showed that stress induced expression of the transgene *PgeIF4A* conferred high level of drought tolerance.

### Characterization of *PgeIF4A* transgenic plants under simulated salt stress

**Figure 6:**
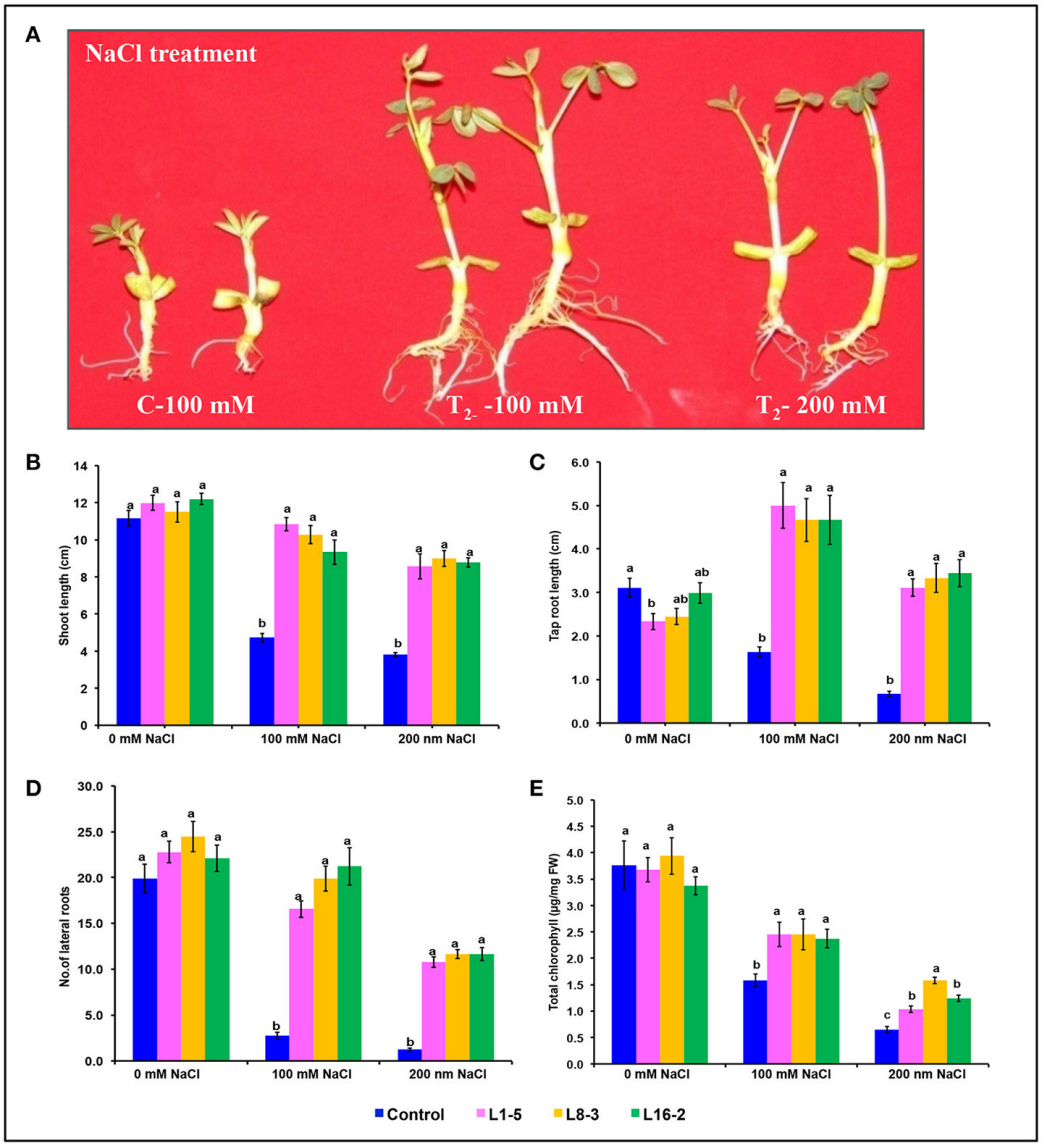
**Characterization of *PgeIF4A* groundnut transgenic plants under simulated salinity stress conditions. (A)** Growth performance of *PgeIF4A* expressing transgenic plants (T_2_) and control on 100 and 200 mM NaCl containing medium. **(B)** Graphical representation of relative shoot length in cm. **(C)** Tap root length in cm. **(D)** Number of lateral roots. **(E)** Chlorophyll retention of transgenic and control plants grown under 0, 100, and 200 mM NaCl medium. All the experiments performed in triplicates and data represented as mean (*n* = three biological triplicates) by using two way ANOVA with LSD and *P* < 0.005. Different alphabets indicated the significant differences between the treatments. Similar letters denotes non significant.

The significant difference was observed in the shoot length, tap root length, and lateral root formation in wild type (C) and transgenic lines (L1-5, L8-3, and L16-2) under both concentrations of NaCl (100 and 200 mM). The shoot and tap root lengths of transgenics were noted as 10.46 and 3.48 cm compared with control plants as 6.57 and 1.8 cm, respectively, under 200 mM NaCl stress condition (Figures 6A–C). Formation of the lateral roots were significantly higher than the control under both concentrations of NaCl (Figure 6D). The growth of the transgenics at 200 mM NaCl were significantly lower for each of the line tested when compared to those at 100 mM (Figure 6; Tables 5, 6). However, the treated transgenic plants recovered very well when transferred to soil under normal growth conditions.

**Table 5:** **Shoot length, tap root length, number of lateral roots, and total chlorophyll content of three transgenic plants and one control were subjected to different concentrations of NaCl (salinity) stress treatments**.

**Traits**	**Components**	**Degree of freedom**	**Sum Square (SS)**	**Mean Square (MS)**	***F*-value**	***P*-value**	**Significance**
Shoot Length (cm)	Treatment (T)	2	440	220	95.3	0.000	^***^
Shoot Length (cm)	Plant lines (L)	3	372.5	124.2	53.8	0.000	^***^
Shoot Length (cm)	LxT interaction	6	136.3	22.7	9.8	0.000	^***^
Tap root length (cm)	Treatment (T)	2	55.3	27.6	21.8	0.000	^***^
Tap root length (cm)	Plant lines (L)	3	83.9	28	22.1	0.000	^***^
Tap root length (cm)	LxT interaction	6	74.3	12.4	9.8	0.000	^***^
No. of lateral roots	Treatment (T)	2	4362.5	2181.3	130.1	0.000	^***^
No. of lateral roots	plant lines (L)	3	2746	915.3	54.6	0.000	^***^
No. of lateral roots	LxT interaction	6	887.6	147.9	8.8	0.000	^***^
Total Chlorophyll	Treatment (T)	2	40.239	20.119	136.92	0.000	^***^
Total Chlorophyll	Plant lines (L)	3	2.005	0.668	4.55	0.012	^*^
Total Chlorophyll	LxT interaction	6	1.488	0.248	1.688	0.167	ns

*Two-way ANOVA was used to determine treatment (T), plant lines (L), and plant lines-by-treatment (LxT) effects*.

* and *** *Indicate significant and highly significant correlation at p < 0.05 and p < 0.01 respectively; ns, non significant*.

**Table 6:** **Shoot length, tap root length, number of lateral roots, and total chlorophyll content of three transgenic plants and one control were subjected to different concentrations of NaCl (salinity) stress treatments**.

**Traits**	**Treatment (mM-NaCl)**	**Treatment mean**	**Significance**
**(A)**			
Shoot length (cm)	0	11.71	A
Shoot length (cm)	100	8.79	B
Shoot length (cm)	200	7.54	C
Tap root LENGTH (cm)	0	3.99	A
Tap root Length (cm)	100	2.72	B
Tap root length (cm)	200	2.64	B
No. of lateral roots	0	22.31	A
No. of lateral roots	100	15.11	B
No. of lateral roots	200	8.83	C
Total chlorophyll	0	3.704	A
Total chlorophyll	100	2.212	B
Total chlorophyll	200	1.125	C
**Traits**	**Plant lines**	**Mean of plants**	**Significance**
**(B)**			
Shoot length (cm)	L1-5	10.46	A
Shoot length (cm)	L8-3	10.26	A
Shoot length (cm)	L16-2	10.10	A
Shoot length (cm)	Control	6.57	B
Tap root length (cm)	L1-5	3.70	A
Tap root length (cm)	L8-3	3.48	A
Tap root length (cm)	L16-2	3.48	A
Tap root length (cm)	Control	1.80	B
No. of lateral roots	L1-5	18.67	A
No. of lateral roots	L8-3	18.33	A
No. of lateral roots	L16-2	16.70	A
No. of lateral roots	Control	7.96	B
Total chlorophyll	L1-5	2.41	AB
Total chlorophyll	L8-3	2.66	A
Total chlorophyll	L16-2	2.33	AB
Total chlorophyll	Control	2.00	B

*Based on Two-way ANOVA results, treatments mean **(A)**, and plant lines data **(B)** were compared by Turkey-Kramer test at 5% LSD. The treatments were significant, different alphabet letters were indicated. Similar letters denotes non-significant*.

In leaf senescence assay, on 2nd day of incubation, the leaf discs from control had bleaching of the chlorophyll as with increasing concentration of NaCl also. However, minimal bleaching was observed in leaf discs from transgenic plants. Total chlorophyll content of these leaf discs after 4 days of stress imposition was retained about 2.3–2.6 μg/mg FW, whereas in control it was observed 2.0 μg/mg FW (Figure 6E). As the concentration of NaCl (100–200 mM) increased the chlorophyll content decreased in accordance with phenotype observed (Figure 6E). The results showed that stress induced expression of the transgene *PgeIF4A* could have conferred high level of salt tolerance.

### *PgeIF4A* transcript analysis by semi quantitative and qRT-PCR

**Figure 7:**
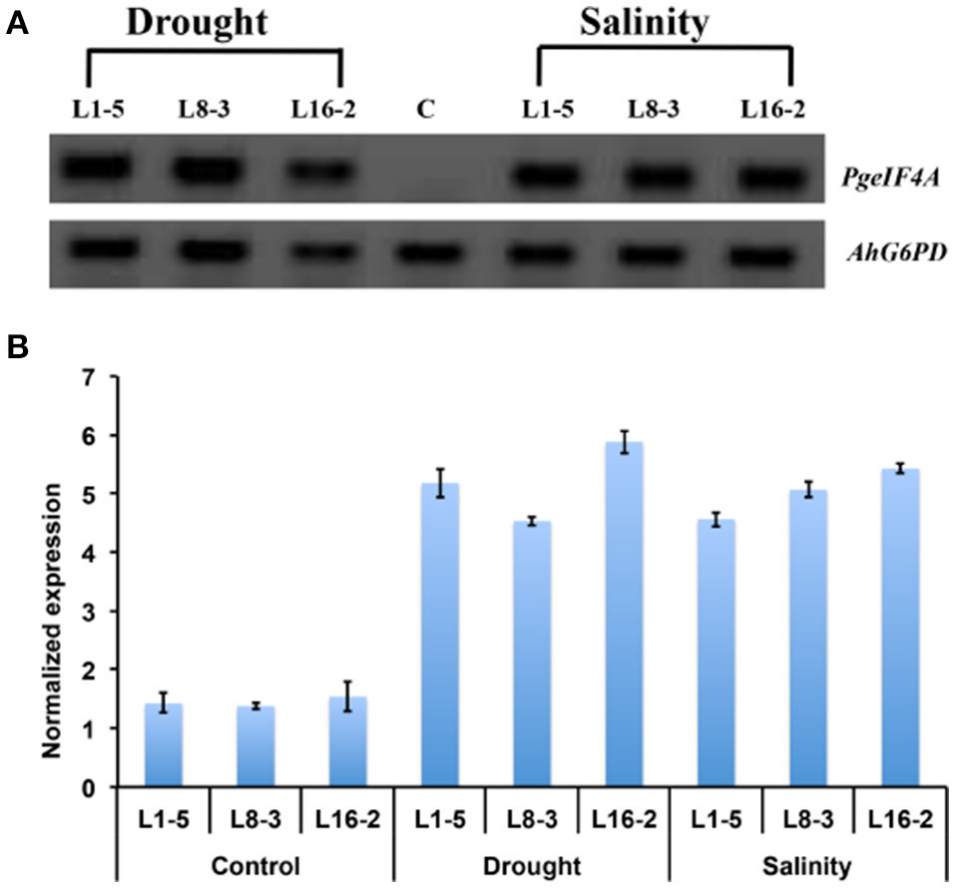
**Expression of *PgeIF4A* transcript in transgenic groundnut (T_2_) lines. (A)** Semi quantitative RT-PCR showing transcript expression pattern of *PgeIF4A* and *G6PD* (*Glucose 6 phosphate 1 dehydrogenase*) in control and transgenic lines grown under mannitol (drought) and NaCl (salinity) stress conditions. **(B)** qRT-PCR expression analysis of transgenic plants under un treated (control) and treated (drought and salinity) conditions. The Ct-values of the samples were normalized with *G6PD* house keeping gene. Lane C: non-transgenic, Lane: L1-5, L8-3, and L16-2 represented T_2_ transgenic lines.

The expression of *PgeIF4A* transcript was confirmed by semi quantitative PCR and qRT-PCR. The *PgeIF4A* transcript accumulated in homozygous T_2_ lines (L1-5, L8-3, and L16-2) grown under stress (mannitol and NaCl) conditions (Figure 7A). The qRT-PCR results showed that the *PgeIF4A* transcript could be detected in all three transgenic plants grown under normal and stress conditions (Figure 7B). The quantitative regulation of *PgeIF4A* in response to drought/salt stress was higher than the plants grown under normal growth conditions. The transgene expression in drought and salinity treated plants was about 4–5-folds higher than the transgenic lines grown under normal conditions (Figure 7B). It indicated that the transgene expression is positively correlated upon stress imposition.

### Biochemical analysis of transgenic plants under salt stress

**Figure 8:**
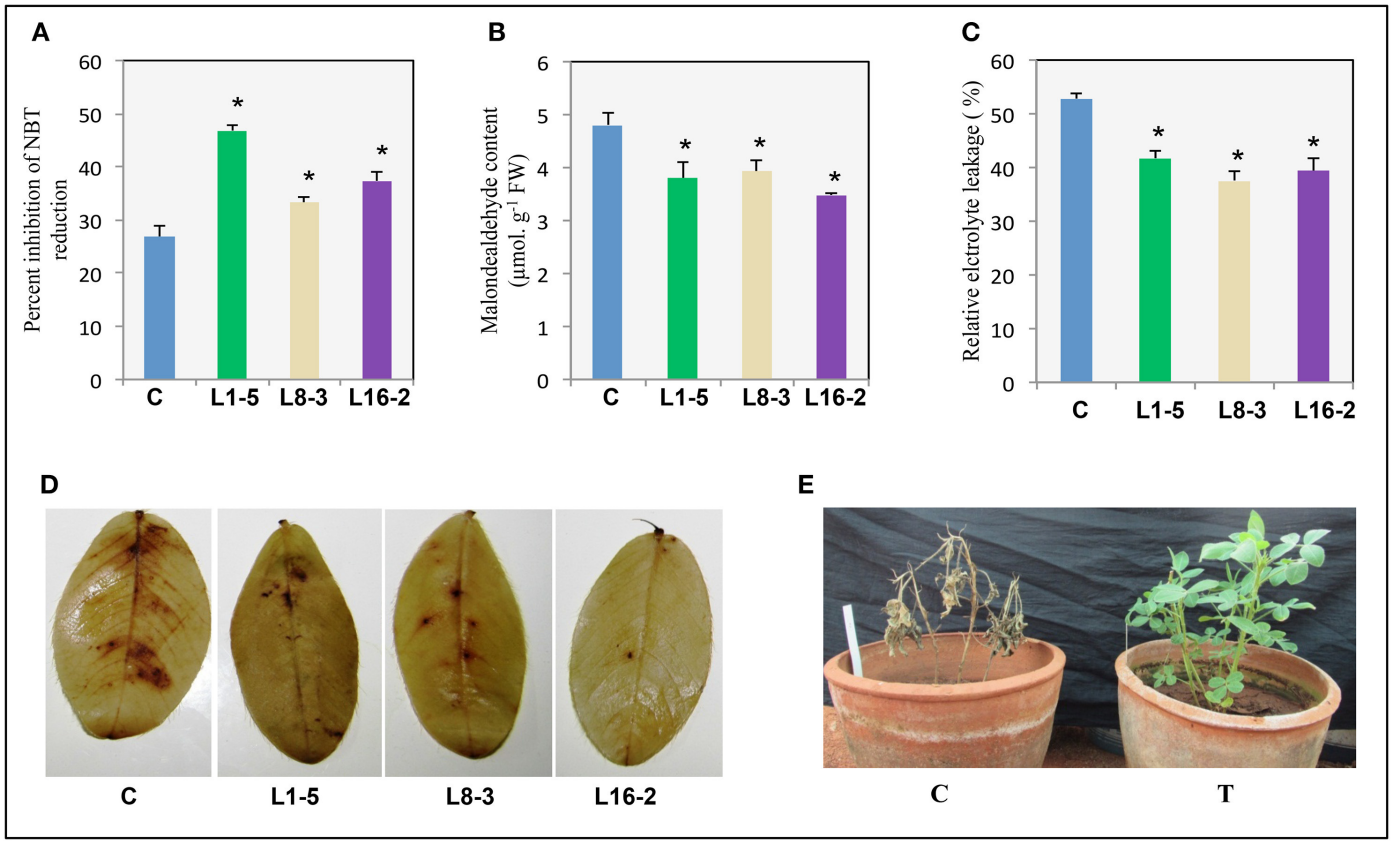
**Response of transgenic plants expressing *PgeIF4A* to salinity stress**. One month old control and transgenic plants were imposed with salinity stress (250 mM NaCl) for 2 weeks. **(A)** SOD activity (% inhibition of NBT). **(B)** Lipid peroxidation (malondialdehyde content). **(C)** Relative electrolyte leakage percentage. **(D)** DAB (3,3′-Diaminobenzidine) staining of WT and transgenic groundnut leaves. **(E)** Control (C) and *PgeIF4A* expressing transgenics (T) allowed to recovery after salinity treatment. Data represented as mean of ±*SE* (*n* = 3) using one way ANOVA. ^*^Denotes significant difference between WT treated and transgenics (L1-5, L8-3, and L16-2) at *p* < 0.05.

The superoxide dismutase (SOD) activity in control and transgeni lines (T_2_) was assessed by measuring th percentage of NBT inhibition. The percentage of NBT inhibition in control was noted as 26.9% compared to the transgenic lines such as L1-5, L8-3, and L16-2 was 46.7, 33.3, and 37.2%, respectively (Figure 8A). It indicated that the superoxide dismutase accumulation was significantly increased, thery by improved the scavenging of reactive oxygen species in *PgeIF4A* expressing transgenic plants. The lipid peroxidation of control and transgenic lines were estimated in leaf tissues by measuring the MDA accumulation through TBARS assay. The higher MDA accumulation was observed in control (4.8 μmol/g FW) compared to transgenic lines L1-5, L8-3, and L16-2 (3.8, 3.9, and 3.4 μmol/g FW in, respectively (Figure 8B). The percentage of ion electrolyte leakage was higher in control (52.8%), compared to transgenic lines L1-5, L8-3, and L16-2 (41.6, 37.4, and 39.4% in, respectively; Figure 8C). It indicated that the electrolyte leakage in transgenics was significantly lower than the control plants, thereby enhanced the membrane stability during stress conditions. Further, 3, 3′ Diaminobenzidin (DAB) analysis detected *in-situ* H_2_O_2_ accumulation. H_2_O_2_ was visually detected by staining the leaves of *PgeIF4A* expressing transgenic and wild type plants under salinity stress (Figure 8D). The reddish brown color produced by DAB in transgenics was visually less compared to control plants, which indicated less H_2_O_2_ production in transgenic plants. Apart from these biochemical parameters, stress induced control plants showed leaf necrosis, bleaching of chlorophyll, and mortality compared to transgenic plants. The transgenic plants recovered completely (Figure 8E). The percentage of salt stress induced oxidative stress damage in L1-5, L8-3, and L16-2 lines was significantly lower than the control plants. The transgenic lines expressing *PgeIF4A* displayed improved membrane stability and RO scavenging capacity.

## Discussion

Eukaryotic translational initiation factor 4A belongs to the family of helicases, important proteins involved in several cellular and metabolic processes including abiotic stress tolerance in plants (Tuteja et al., 2014). Transgenic approaches have been exploited for expression of helicases from different species in tobacco, Arabidopsis, rice etc, conferred abiotic stress tolerance (Sanan-Mishra et al., 2005; Luo et al., 2009; Gill et al., 2013). Groundnut is one of important leguminous crops growing in semi arid and tropical regions. In current study, we have successfully developed transgenic groundnut *cv* JL24 by transferring *PgeIF4A* gene driven by stress inducible *rd29A* promoter. The DECs used as explants, since they were reported as explants having more regeneration capacity than any other explant in groundnut. The cut surfaces of DEC were acted as competent cells for the *Agrobacterium* infection and more amenable for multiple shoot proliferation from individual shoot (Tiwari and Tuli, 2012). Recovery of transformed multiple shoots was higher in DEC compared to immature embryos (Mehta et al., 2013). The putative transformed plants showed an amplification of both cassettes *rd29A-PgeIF4A* and *nos-bar* with 11% transformation efficiency. The transformation efficiency varied based on the different explants and genotypes, growth regulators used for generating transgenic plants (Mallikarjuna et al., 2016).

The transformed plants survived and displayed normal growth without any growth penalty under non stress and stress conditions. It could be due to stress inducible expression of the *PgeIF4A* gene in presence of *rd29A* promoter. The expression of *OsDREB2A* under the regulation of *rd29A* promoter conferred drought and salinity tolerance and minimized the negative growth effects under stress and non-stress condition in rice (Mallikarjuna et al., 2011). However, constitutively expressing *AtDREB1A* groundnut plants showed stunted growth and phenotypic abnormality when compared with those transgenics with the same gene under *rd29A* inducible expression (Bhatnagar-Mathur et al., 2007; Sarkar et al., 2016). Similarly, constitutive over expression of *AtDREB1A* gene caused growth retardation in transgenic *Arabidopsis* plants (Kasuga et al., 1999). The *PgeIF4A* expressing groundnut transgenic lines showed enhanced root and shoot growth over the control under stress conditions. Since the root system developed strongly, access to the uptake of nutrients could be increased in the plants. *DREB1A* expressing groundnut plants had better access of water uptake since the root system penetrated into deeper soil which resulted in significant tolerance under limited moisture conditions (Bhatnagar-Mathur et al., 2014; Sarkar et al., 2016).

The transgenic lines showed the accumulation of transgene transcript in three homozygous T_2_ lines grown under drought and salinity conditions. The qRT-PCR data showed that the expression of transgene was induced in three transgenic lines under drought and salt stress conditions. It could be due to the positive regulation of *rd29A* promoter which involved in stress inducible expression of transgene. Similarly, higher expression of *AtDREB1A* was seen in groundnut transgenic plants which were regulated by *rd29A* promoter (Sarkar et al., 2014, 2016). It indicated that there was positive correlation between regulation of transgene and stress tolerance. Similar kinds of results have been reported with *mtlD, AtDREB1A* expressing groundnut lines also (Bhauso et al., 2014; Sarkar et al., 2016).

The role of *PgeIF4A* gene at T_2_ generation was assessed by physiological and biochemical analysis. The number of branches and leaf area in transgenic plants increased better than control plants. Moreover, the thickness of the branches and shoots increased enormously in transformed plants. The shoot length, tap root length, and number of later roots of L1-5 and L8-3 significantly higher than L16-2 line at lower and higher concentrations of mannitol (Figures 5C–E). The shoot and taproot length of L1-5 line exhibited higher than other transgenic lines at lower and higher concentrations of mannitol and 100 mM NaCl. But at higher concentration of NaCl these lines showed similar performance (Figure 6). Interestingly, when tap root length increased, number of lateral root formation reduced under lower concentration of mannitol and NaCl. The tap root system was very well-developed with more robustness and well-adopted root hair in the transgenic plants. Moreover, the treated transgenic plants recovered very well-upon transferred them into soil.

In addition, the tap root of all transgenic lines showed higher growth under 100 mM than the plants grown under normal growth conditions. The transgenic lines L8-3 retained more chlorophyll than other two lines under both concentrations of mannitol and NaCl. However, at 200 mM NaCl there was a significant difference in chlorophyll retention between transgenic lines. The transgenic plants performed well without growth penalty which signifying that the plants were not been stunted due the regulation of stress induced *rd29A* promoter. The transgenic line L16-2 did not performed as like other two lines, this could be due to integration of two copies of transgenes in the genome. However, there was no much significance difference observed between transgenic lines and gene silencing was not occurred in L16-2 line. The transgenic plantlets expressing *PDH45* exhibited higher survival rate, shoot and root growth in comparison with wild type plants under mannitol and salt stress conditions. The improved lateral root formation could be due to expression of related target genes under stress conditions (Manjulatha et al., 2014).

The membrane damage leads to accumulation of high MDA, electrolyte leakage and reduction in NBT inhibition levels in cells. Transgenic line L1-5 expressing *PgeIF4A* showed increased in NBT inhibition levels and less MDA, minimal electrolyte leakage which coupled with higher chlorophyll retention under stress conditions (Figure 8). However, there was no much significance difference in these parameters when compared with all transgenic lines. The cell membrane stability was enhanced by reducing the electrolyte leakage in transgenic plants. The transgenic plants possessed efficient anti oxidative machinery which protected the plant cells from ROS induced oxidative damage. In other studies also increase in level of oxidative markers were reported when heterologous genes expressed in groundnut such as *AtNHX* (Asif et al., 2011), *PHD45* (Manjulatha et al., 2014), *AtNAC2* (Patil et al., 2014), *MuNAC4* (Pandurangaiah et al., 2014).

*PgeIF4A* expressing transgenic lines (T_2_) sensed the stress by way of coordinating with oxidative enzyme machinery. As a result high RO scavenging, more retention of chlorophyll in transgenic lines was noted and there by protected the plants by growing normally under stress and non-stress conditions. The improved JL-24 variety exhibited superior growth parameters under simulated stress condition. The plants showed tolerance to herbicide which could be additional feature to control the weeds. The *eIF4A* gene from *P. glaucum* played a key role in stress tolerance and these plants will be further validated under field conditions.

## Conclusion

Groundnut *cv* JL-24 is an important high yielding and drought sensitive variety was used for improving drought and salt stress tolerant by transferring *PgeIF4A* gene through transgenic approach. In present study, three lines clearly showed drought, salt, and oxidative stress tolerance under simulated conditions. Among these lines, L1-5 exhibited super growth performance and scavenging free radicals under salt stress conditions. *PgeIF4A* expression in groundnut was strongly associated with enhancement of growth parameters and chlorophyll retention. This strong regulation could be due to firm regulated expression of *PgeIF4A* gene controlled by stress inducible *rd29A* promoter, which leads to the stress tolerance in subsequent generations.

## Author contributions

TS and GM: Planned and executed the experiments; JV: biochemical analysis, PS and MR: provided gene construct GM, TS, JV, and PS: Prepared manuscript.

## Funding

Financial support received from Rashtriya Krishi Vikas Yojana (RKVY), Government of Andhra Pradesh.

### Conflict of interest statement

The authors declare that the research was conducted in the absence of any commercial or financial relationships that could be construed as a potential conflict of interest.
