# Supplementary material for: Expression of Pennisetum glaucum Eukaryotic Translational Initiation Factor 4A (PgeIF4A) Confers Improved Drought, Salinity, and Oxidative Stress Tolerance in Groundnut
Source: Front Plant Sci. 2017 Apr 7;8:453. doi: 10.3389/fpls.2017.00453 (PMC5383670; doi:10.3389/fpls.2017.00453)
Supplement: Supplementary file 1 [file Table1.doc]

**Supplementary Table 1** List of primers used for various aspects of the present study

| **S. No** | **Gene/Cassette name** | **Primer name** | **Sequence** | **role** |
| --- | --- | --- | --- | --- |
| 1 | *rd29A* | rd29A F-*Kpn* I | GGTACCCGACTCAAAACAAACTTACGA | Cloning |
|  |  | rd29A R-*Nde* I | CATATGCATATTTGTGAGTAAAACAGAGG |  |
| 2 | *eIF4A* | PgeIF4AF-*Nde* I | CATATGATGGCGGCGGCCACCACCTC |  |
|  |  | PgeIF4A R-*Not* I | GCGGCCGCGAAGTCCAGCTGCCAGAAAC |  |
| 3 | *nos-bar* | nos F1 | AACCACTACATCCAGACA | Transgene detection |
|  |  | bar R1 | GAAGTCCAGCTGCCAGAAAC |  |
| 4 | *rd29A-eIF4A* | rd29A F1 | AACAGCCACACGACGTAAAC |  |
|  |  | PgeIF4A R1 | TCTCACTCACCGTATGCCAG |  |
| 5 | *PgeIF4A* | RT-PgeIF4A F  RT-PgeIF4A R | CAAGTTCATGACCGAACCAG  TGGGTGATGGTCAACGTATC | quantitative RT-PCR |
| 6 | *G6PD* | Ah_G6PD F  Ah_G6PD R | ACCATTCCAGAGGCTTATGAGC  AAGGGAGTGACTTGAACTCTCC |  |
